# The association between dietary approaches to stop hypertension diet and bone mineral density in US adults: evidence from the National Health and Nutrition Examination Survey (2011–2018)

**DOI:** 10.1038/s41598-023-50423-7

**Published:** 2023-12-27

**Authors:** Xiang-Long Zhai, Mo-Yao Tan, Gao-Peng Wang, Si-Xuan Zhu, Qi-Chen Shu

**Affiliations:** 1 Chengdu Integrated TCM and Western Medicine Hospital, Chengdu, Sichuan China; 2 Chengdu University of Traditional Chinese Medicine, Chengdu, Sichuan China

**Keywords:** Diseases, Gastroenterology, Risk factors

## Abstract

This study aimed to investigate the relationship between the dietary approaches to stop hypertension (DASH) dietary patterns and bone mineral density (BMD) in adults residing in the United States. To achieve this, data from the National Health and Nutrition Examination Survey (NHANES) database for 2011–2018 were utilized. This study utilized the NHANES database from 2011 to 2018, with a sample size of 8,486 US adults, to investigate the relationship between the DASH diet and BMD. The DASH diet was assessed based on nine target nutrients: total fat, saturated fat, protein, fiber, cholesterol, calcium, magnesium, sodium and potassium. The primary outcome measures were BMD values at the total BMD, thoracic spine, lumbar spine, and pelvis. Multivariable linear models were employed to analyze the association between the DASH diet and BMD. Interaction tests, subgroup, and sensitivity analysis were also followed. A negative correlation was observed between the DASH diet and total BMD (OR: − 0.003 [95%CI: − 0.005, − 0.001), pelvic (OR: − 0.005 [95%CI: − 0.007, − 0.002]), and thoracic BMD (OR: − 0.003 [95%CI: − 0.005, − 0.001]). However, the DASH diet does not appear to have a particular effect on lumbar spine BMD (OR: − 0.002 [95%CI: − 0.004, 0.001]). Similarly, when the DASH diet was categorized into tertiles groups, the relationship with total BMD, pelvic BMD, thoracic BMD, and lumbar spine BMD remained consistent. Furthermore, we performed a sensitivity analysis by converting BMD to Z-scores, and the results remained unchanged. Subgroup analyses and interaction tests indicated no significant dependence of BMI, gender, smoking, hypertension, and diabetes on the observed association (all *p* for interactions > 0.05). The DASH diet has been identified as potentially reducing total BMD, while specifically impacting thoracic and pelvic BMD. However, it appears to have no significant effect on lumbar spine BMD.

## Introduction

Bone mineral density (BMD) is a crucial determinant of bone fragility as it represents the amount of bone mineral within bone tissue^1^. Healthy adult BMD typically falls within the range of approximately 1.045 ± 0.135 g/cm^2^ for males and 0.991 ± 0.107 g/cm^2^ for females^2^. The NHANES website also provides assessed BMD status for the U.S. population, for example, with BMD quartiles ranging from 1.143–1.280 g/cm^2^ for 20–29 years males and 1.060–1.171 g/cm^2^ for females of the same age^3^. When BMD drops below a certain threshold, osteoporosis is triggered^4^. In the United States, it is estimated that nearly half of individuals aged 46 and older have low BMD, with projections indicating a rise to over 3 million fractures and an annual cost of $25.3 billion due to osteoporosis by 2025^5^. Moreover, it is anticipated that by 2030, over 70 million Americans will be diagnosed with osteoporosis^6^. Given the aging global population, this condition is recognized as a significant public health concern^7^. Numerous studies have consistently established a strong association between dietary patterns and bone health^8–10^. Adopting a healthy dietary pattern has the potential to impact BMD positively. In their investigation, Hsu E. et al. explored the correlation between plant-based diets and BMD, proposing mechanisms that promote bone health^11^. Recent studies have also indicated that adherence to the Mediterranean diet may be a preventive measure against osteoporosis^12^. Furthermore, a meta-analysis has proved that incorporating soy isoflavones, enriched with omega-3 fatty acids, into dietary supplementation effectively improves women's bone health during menopause^13^. This intervention not only mitigates bone loss caused by menopause but also enhances bone formation while reducing bone resorption^14^.

The Dietary Approaches to Stop Hypertension (DASH) diet, which encompasses reduced sodium and calorie intake along with a diet abundant in fruits, vegetables, low-fat dairy products, whole grains, poultry, fish, nuts, and unsaturated vegetable oils, has received endorsement from the United States Department of Agriculture's Dietary Guidelines for Americans (2020–2025)^15,16^. Additionally, the DASH diet has been found to have various other applications. For instance, Zhang et al.^17^ discovered that the DASH diet can effectively decrease both blood pressure and the incidence of osteoarthritis. Additionally, the DASH diet has been observed to have a glycemic control effect in diabetic patients^18^. Furthermore, a study investigating the relationship between the DASH diet and serum uric acid levels over time revealed that adherence to the DASH diet can reduce serum uric acid levels^19^. Therefore, research on the uses of the DASH diet should include more than just lowering high blood pressure. Considering the rising prevalence of the DASH diet in special populations everyday lives, even the smallest effects that accrue over time could have a substantial impact on our bodies. Thus, it is essential to investigate and give attention to the potential impact of this diet on BMD.

Previous research suggests that the DASH diet may decrease BMD. Recent findings indicated that moderate increases in total fat, fiber intake, and magnesium intake might improve BMD^20–22^. However, a study discovered that the individuals in their study who followed the DASH diet did not meet the recommended values for total fat, fiber intake, and magnesium intake^23^. Additionally, certain studies have shown that adhering to the DASH diet is associated with lower levels of dietary triglycerides (TG) and low-density lipoprotein cholesterol (LDL-C)^24^, both of which are positively associated with BMD^25,26^. Therefore, there may be a risk of indirectly decreasing BMD by implementing the DASH diet.

Given the extensive acceptance of this dietary pattern among the general population, it is crucial to investigate its influence on BMD. We conducted a comprehensive study using NHANES data from 2011 to 2018 to further explore the relationship between the DASH diet and BMD. Our approach involved multivariate linear regression, subgroup analysis, sensitivity analyses, and interaction tests.

## Methods

### Data available

The National Health and Nutrition Examination Survey (NHANES), sponsored by the National Center for Health Statistics (NCHS) within the Centers for Disease Control and Prevention (CDC), is a recurring, nationwide cross-sectional survey. It has been conducted periodically since the 1960s with the primary objective of evaluating the health and nutritional status of both children and adults in the United States. Annually, approximately 5,000 participants are recruited using a multistage stratified sampling method, ensuring a nationally representative sample from counties across the United States. Demographic information and lifestyle data including dietary habits are gathered through surveys and physical examinations. The results of this comprehensive survey are published biennially. Details about survey design and data files can be accessed publicly at https://www.cdc.gov/nchs/nhanes/. The ethics protocol has been formally approved by the Research Ethics Review Board of NCHS, and informed consent was signed by all recruited participants^27^. Notably, this study diligently adheres to the principles delineated by Strengthening the Reporting of Observational Studies in Epidemiology (STROBE) regarding cross-sectional studies^28^.

### Study population

**Figure 1:**
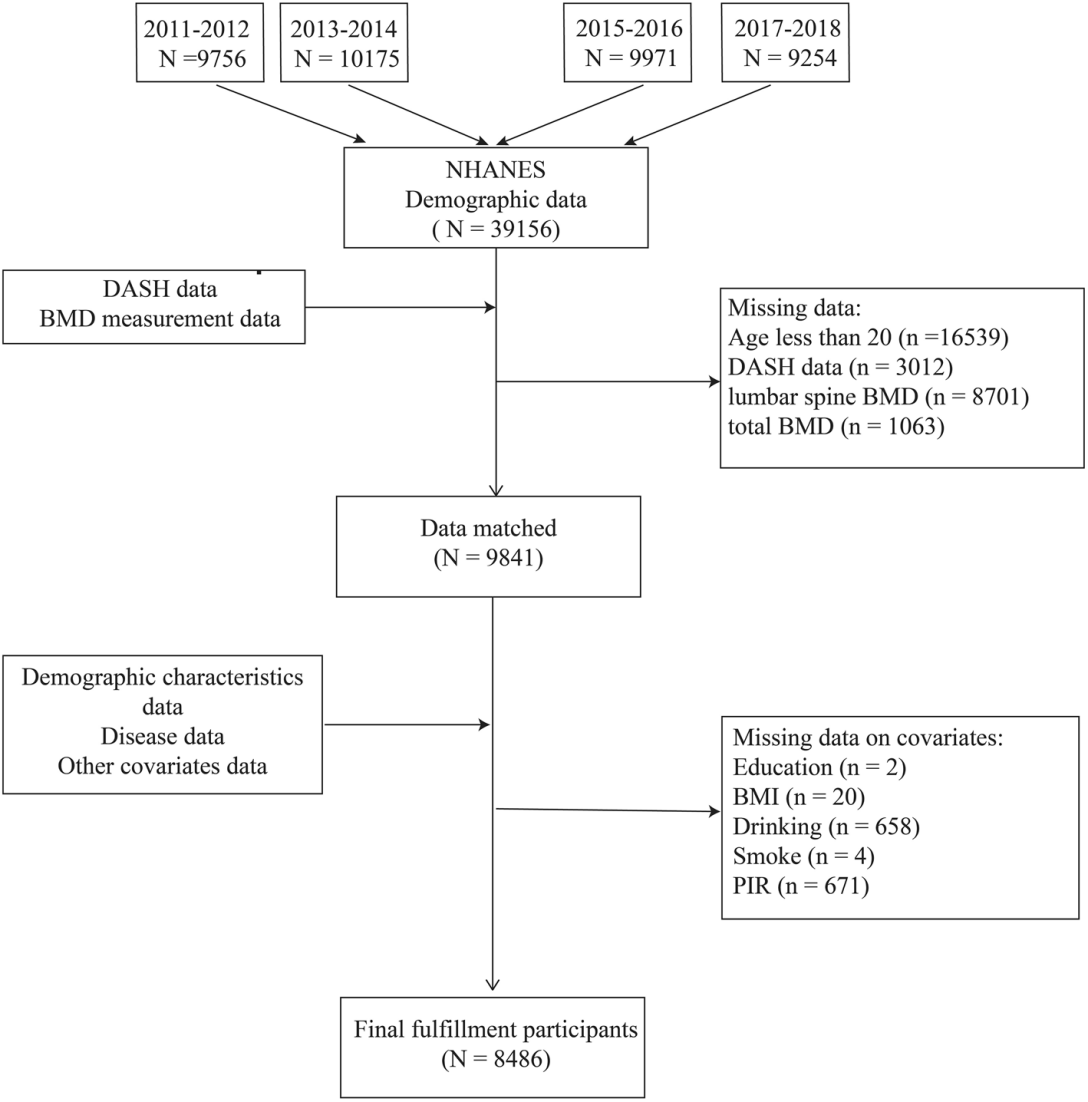
Flowchart of the sample selection from the National Health and Nutrition Examination Survey (NHANES).

This study utilized data from four survey cycles conducted by NHANES (2011–2012, 2013–2014, 2015–2016, and 2017–2018). These specific cycles were selected due to their inclusion of data on total BMD, spanning 2011 to 2018. After a rigorous selection process, the study incorporated a total of 39,156 participants over four biennial periods. The distribution was as follows: 9756 participants from 2011–2012, 10,175 from 2013–2014, 9971 from 2015–2016, and 9254 from 2017–2018. Individuals under the age of 20 were subsequently excluded, which accounted for 16,539 of the initial cohort. Furthermore, we eliminated records that lacked DASH diet scores or BMD data, amounting to 12,776 participants. Additional data pruning included the exclusion of entries missing essential covariates such as education (n = 2), body mass index (BMI) (n = 20), smoking status (n = 4), alcohol consumption (n = 658), and poverty income ratio (PIR) (n = 671). Ultimately, this rigorous process yielded a final sample of 8,486 subjects eligible for analysis, as depicted in Fig. 1.

### Definition of DASH

The DASH diet is a commonly followed dietary regimen incorporating a range of vital nutrients, including total fat, saturated fat, protein, fiber, cholesterol, calcium, magnesium, sodium and potassium. A detailed elucidation on the calculation of the DASH diet has been previously recorded^23^ and can be accessed in Supplementary Material 2 for additional information.

### BMD measurement

Dual-energy X-ray absorptiometry (DXA), a widely acknowledged and extensively employed bone densitometry technique in contemporary clinical practice, offers significant merits, including expeditiousness, ease of use, and limited radiation exposure. The Hologic Discovery model A densitometer manufactured by Hologic, Inc., based in Bedford, MA, USA, was utilized for conducting the scans. The BMD measurements were performed meticulously and professionally by radiologists with training and certification. To maintain result accuracy, individuals who were pregnant, had recently used contrast media, or were overweight were excluded from the study. For more comprehensive information on the BMD measurements and the protocols employed, the NHANES website provides detailed documentation.

### Covariates

We utilized multivariable adjustment models to address the possibility of confounding variables in the correlation between DASH and BMD, as previously employed in related studies^29,30^. The demographic variables examined in our study encompass gender (male/female), age (in years), ethnicity (Mexican American/Non-Hispanic white/Non-Hispanic black/Other races), educational level (less Than 9th grade/9-11th grade (includes 12th grade without diploma)/High School Graduate/GED or Equivalent/Some College or AA Degree/College Graduate or above), marital status (married/widowed/divorced/separated/never married/living with partner) and PIR (low-income/middle-income/high-income)^31^. Additionally, the study considers smoking habits (never/former/current) and patterns of alcohol consumption (never/former/heavy/mild/moderate) as outlined in an earlier report^32^. In addition, the research incorporates anthropometric and laboratory covariates, namely BMI (kg/m^2^), which is determined by dividing weight in kilograms by the square of height in meters. Health status variables encompass hypertension (Yes/No) and diabetes (Yes/No). Diabetes is defined as: (1) doctor told you have diabetes, (2) glycohemoglobin HbA1c(%) >  = 6.5, (3) fasting glucose (mmol/l) >  = 7.0, (4) random blood glucose (mmol/l) >  = 11.1, (5) two-hour OGTT blood glucose (mmol/l) >  = 11.1, or (6) use of diabetes medication or insulin^33^. Hypertension is defined as: taking antihypertensive medication, a doctor's diagnosis of hypertension, or having systolic blood pressure ≥ 140 mmHg or diastolic blood pressure ≥ 90 mmHg on three consecutive readings^34^.

### Statistical analysis

In our study, we adhered rigorously to the statistical analysis protocols endorsed by the CDC. Additionally, we considered the intricacies inherent in a complex multistage cluster survey design during our analytical procedures^35,36^. Mean values accompanied by standard errors were used to represent continuous variables, while percentages were employed for categorical variables. Subsequently, we employed a Student's t-test (for continuous variables) or a chi-square test (for categorical variables) to evaluate group disparities. Linear regression was applied to estimate the relationship between DASH diet and BMD. DASH diet served as the dependent variable and was modeled both categorically (in three categories) and continuously (scores), respectively. Meanwhile, BMD, as the independent variable, was modeled continuously, both in its raw scale and Z-score form. Estimated effects size was presented as betas (βs) along with their respective 95% confidence intervals (CIs). On one hand, our primary objective was to assess the impact of DASH dietary differences among participants on BMD. To achieve this, we categorized DASH scores into tertiles, allowing us to compare the BMD of populations in the highest tertile with those in the lowest tertile. On the other hand, we also conducted analyses using its continuous scale of the DASH scores, enabling us to explore its potential linear relationships with BMD. We accounted for potential confounding variables. Model 1 did not incorporate any additional factors. Model 2, on the other hand, included adjustments for age, sex, and race. Model 3 entailed adjustments for sex, age, race, education level, PIR, marital status, BMI, smoking status, diabetes, alcohol consumption, and hypertension. Subgroup analyses were conducted based on age, sex, BMI, smoking, hypertension, and diabetes status. Additionally, subgroup analyses as well as interaction tests were conducted to assess potential modifying effects of these variables on the relationship between DASH and BMD, and potential interactions between these variables and DASH. A significance level of less than 0.05 is commonly considered to indicate statistical significance in statistical analysis. The statistical analyses conducted in this study were executed utilizing R software (version 4.1.2; http://www.R-project.org, R Foundation for Statistical Computing, Vienna, Austria).

### Ethics approval and consent to participate

The ethics review board of the National Center for Health Statistics approved all NHANES protocols.

## Results

### Essential characteristics of the included participants

Table 1 presents the pertinent details regarding the inclusion of 8,486 participants. The mean age of the included population was 39.07 ± 0.28 years, with males accounting for 51.82% and females accounting for 48.18%. Measurements of total BMD, thoracic spine BMD, lumbar spine BMD, and pelvic BMD yielded values of 1.11 ± 0.01 g/cm^2^, 0.82 ± 0.01 g/cm^2^, 1.04 ± 0.01 g/cm^2^, and 1.25 ± 0.01 g/cm^2^, respectively. The clinical characteristics of the participants, stratified by DASH tertiles, are presented in Table 2. This table demonstrates notable disparities in variables including age, gender, race, BMI, hypertension, education, pelvic BMD, thoracic spine BMD, and total BMD among the three DASH tertiles (all p-values < 0.05, refer to Table 2 for specific data). Our study revealed that individuals with higher DASH levels exhibited a higher likelihood of being female, older, and of Mexican American or other racial backgrounds, with these associations achieving statistical significance at p-value < 0.05 (refer to Table 2 for specific data).

**Table 1:** Baseline characteristics of participants.

Characteristics	Means (standard error) or percentage
Age (year)	39.07 (0.28)
Body mass index (kg/m^2^)	28.78 (0.16)
DASH	2.30 (0.03)
LumbarSpineBMD (g/cm^2^)	1.04 (0.01)
PelvicBMD (g/cm^2^)	1.25 (0.01)
ThoracicSpineBMD (g/cm^2^)	0.82 (0.01)
Total BMD (g/cm^2^)	1.11 (0.01)
Sex (%)	
Male	51.82
Female	48.18
Race (%)	
Mexican American	9.85
Non-Hispanic White	63.40
Non-Hispanic Black	10.79
Other Race	15.97
Education level (%)	
Less than 9th grade	3.16
9–11th grade (Includes 12th grade with no diploma)	8.54
High school graduate /GED or equivalent	21.66
Some college or AA degree	33.13
College graduate or above	33.51
Marital status (%)	
Married	51.28
Widowed	1.12
Divorced	9.20
Separated	2.55
Never married	25.18
Living with partner	10.67
Smoking status (%)	
Never	60.23
Former	19.26
Now	20.50
PIR (%)	
Low-income	15.55
Middle-income	48.20
High-income	36.25
Hypertension (%)	
Yes	25.90
No	74.10
Diabetes (%)	
Yes	26.33
No	73.67
Alcohol (%)	
Former	8.11
Heavy	27.21
Mild	34.61
Moderate	20.76
Never	9.31

Notes: All values are presented as proportion (%), or mean(standard error).

PIR, ratio of family income to poverty; BMD, bone mineral density.

**Table 2:** Baseline characteristics of participants based on DASH diet tertiles.

Dietary approaches to stop hypertension	Tertile 1	Tertile 2	Tertile 3	p for trend
Age (year)	38.68 (0.30)	39.65 (0.36)	42.33 (0.99)	** < 0.001**
Body mass index (kg/m^2^)	29.15 (0.17)	28.09 (0.21)	27.70 (0.60)	** < 0.001**
LumbarSpineBMD (g/cm^2^)	1.04 (0.01)	1.03 (0.01)	1.03 (0.01)	0.120
PelvicBMD (g/cm^2^)	1.26 (0.01)	1.24 (0.01)	1.20 (0.01)	** < 0.001**
ThoracicSpineBMD (g/cm^2^)	0.83 (0.01)	0.81 (0.01)	0.80 (0.01)	** < 0.001**
Total BMD (g/cm^2^)	1.12 (0.01)	1.11 (0.01)	1.10 (0.01)	** < 0.001**
Sex (%)				** < 0.001**
Male	53.96	48.28	38.17	
Female	46.04	51.72	61.83	
Race (%)				**< 0.001**
Mexican American	9.50	10.29	13.62	
Non-Hispanic White	64.04	62.44	57.98	
Non-Hispanic Black	12.22	7.88	8.46	
Other Race	14.24	19.39	19.94	
Education level (%)				**< 0.001**
Less than 9th grade	2.70	4.02	4.64	
9–11th grade (Includes 12th grade with no diploma) 9.23		7.44	3.81	
High school graduate/GED or equivalent	22.82	19.78	14.03	
Some college or AA degree	34.44	30.83	26.57	
College graduate or above	30.82	37.93	50.95	
Marital status (%)				0.250
Married	50.73	52.37	52.47	
Widowed	1.27	0.75	1.61	
Divorced	9.14	9.19	11.07	
Separated	2.29	3.24	1.02	
Never married	25.69	24.02	25.92	
Living with partner	10.88	10.44	7.91	
Smoking status (%)				0.110
Never	58.74	59.11	70.42	
Former	19.51	20.04	18.01	
Now	21.75	20.86	11.57	
PIR (%)				**0.010**
Low-income	15.83	15.18	12.47	
Middle-income	49.58	45.46	45.27	
High-income	34.59	39.36	42.26	
Hypertension (%)				**0.040**
Yes	27.28	24.18	27.82	
No	72.72	75.82	72.18	
Diabetes (%)			0.070	
Yes	9.00	7.67	6.63	
No	91.00	92.33	93.37	
Alcohol (%)				**0.01**
Former	8.10	8.03	9.12	
Heavy	27.59	26.98	19.59	
Mild	25.28	33.14	35.02	
Moderate	20.83	20.72	19.33	
Never	8.20	11.13	16.94	

Significant values are in [bold].

Notes: All values are presented as proportion (%), or mean(standard error).

PIR, ratio of family income to poverty; BMD, bone mineral density.

### The association between DASH and BMD

Table 3 presents the results of a linear regression analysis examining the correlation between DASH and BMD. The fully adjusted models revealed significant negative associations between DASH and total BMD (β = − 0.003, 95% CI: − 0.005, − 0.001), pelvic BMD (β = − 0.005, 95% CI: − 0.007, − 0.002), and thoracic spine BMD (β = − 0.003, 95% CI: − 0.005, − 0.001). Taking the effect size of DASH on total BMD (β = − 0.003) as an example, it suggested that for every one-point increase in DASH score, there is a corresponding decrease of 0.003 g/cm^2^ in total BMD. However, the relationship between the DASH diet and lumbar spine BMD did not achieve statistical significance (β = − 0.002, 95% CI: − 0.004, 0.001). We also performed a sensitivity analysis by transforming DASH from a continuous to a categorical variable (tertiles), and the outcomes remained consistent.

**Table 3:** Multivariate linear regression analysis of the association between the DASH diet and bone mineral density.

Total bone mineral density (g/cm^2^)	Model 1^a^	Model 2^b^	Model 3^c^
Dietary approaches to stop hypertension	-0.006 (− 0.007, − 0.004) < **0.001**	− 0.002 (− 0.004, − 0.000) < **0.001**	− 0.003 (− 0.005, − 0.001) **0.004**
Q1	**Reference**	**Reference**	**Reference**
Q2	− 0.011 (− 0.018, − 0.005) < **0.001**	− 0.003 (− 0.009, 0.003) 0.320	− 0.004 (− 0.011, 0.002) 0.170
Q3	− 0.020 (− 0.037, − 0.003) **0.020**	− 0.002 (− 0.020,0.015) 0.770	− 0.009 (− 0.024, 0.007) 0.270

Lumbar spine-BMD (g/cm^2^)	Model 1^a^	Model 2^b^	Model 3^c^
Dietary approaches to stop hypertension	− 0.004 (-0.006, -0.002) **0.002**	-0.002 (-0.004, 0.000) 0.100	-0.002 (-0.004, 0.001) 0.136
Q1	**Reference**	**Reference**	**Reference**
Q2	− 0.008 (− 0.017, 0.000) **0.046**	− 0.003 (− 0.012, 0.005) 0.424	− 0.003 (− 0.012, 0.006) 0.471
Q3	− 0.006 (− 0.032, 0.020) 0.646	0.002 (− 0.023, 0.027) 0.851	0.003 (− 0.022, 0.027) 0.831

Thoracic spine-BMD (g/cm^2^)	Model 1^a^	Model 2^b^	Model 3^c^
Dietary approaches to stop hypertension	− 0.007 (− 0.009, − 0.005)** < 0.001**	− 0.005 (− 0.007, − 0.003) < **0.001**	− 0.003 (− 0.005, − 0.001) **0.003**
Q1	**Reference**	**Reference**	**Reference**
Q2	− 0.015 (− 0.021, − 0.009) < **0.001**	− 0.010 (− 0.016, − 0.004) **0.001**	− 0.008 (− 0.014, − 0.001) **0.018**
Q3	− 0.022 (− 0.042, − 0.003) **0.027**	− 0.015 (− 0.034, 0.004) 0.123	− 0.014 (− 0.031, 0.003) 0.100

Pelvic-BMD (g/cm^2^)	Model 1^a^	Model 2^b^	Model 3^c^
Dietary approaches to stop hypertension	− 0.010 (− 0.013, − 0.008) < **0.001**	− 0.007 (− 0.009, − 0.004) < **0.001**	− 0.005 (− 0.007, − 0.002) < **0.001**
Q1	**Reference**	**Reference**	**Reference**
Q2	− 0.018 (− 0.028, 0.008) < **0.001**	− 0.008 (− 0.018, 0.001) 0.090	− 0.004 (− 0.014, 0.006) 0.426
Q3	− 0.048 (− 0.041, − 0.001) **0.037**	− 0.029 (− 0.050, − 0.007) **0.011**	− 0.021 (− 0.040, − 0.001) **0.037**

Significant values are in [bold].

Note: BMD values are presented as raw variables without any transformation.

Model 1^a^: no covariates were adjusted; Model 2^b^: adjusted for sex, age, and race; Model 3^c^: adjusted for age, race, sex, education, ratio of family income to poverty, marital status, body mass index, alcohol intake, smoking status, diabetes, and hypertension.

BMD, bone mineral density; 95% CI, 95% confidence interval.

**Table 4:** DASH diet and bone mineral density correlationa Z-score linear regression analysis.

Total bone mineral density (g/cm^2^)	Model 1^a^	Model 2^b^	Model 3^c^
Dietary approaches to stop hypertension	− 0.05 (− 0.068, − 0.035) < **0.001**	− 0.018 (− 0.033, − 0.003)** 0.020**	− 0.026 (− 0.044, − 0.008) ** 0.005**
Q1	**Reference**	**Reference**	**Reference**
Q2	− 0.103 (− 0.162, − 0.045) < **0.001**	− 0.028 (− 0.083, 0.028) 0.320	− 0.04 (− 0.090, 0.020) 0.100
Q3	− 0.039 (− 0.200, 0.120) 0.640	− 0.023 (− 0.181, 0.136) 0.770	− 0.07 (− 0.221, 0.067) 0.120

Lumbar spine-BMD (g/cm^2^)	Model 1^a^	Model 2^b^	Model 3^c^
Dietary approaches to stop hypertension	− 0.025 (− 0.040, − 0.010) **0.002**	− 0.013 (− 0.029, − 0.003) 0.106	− 0.012 (− 0.029, 0.004) 0.136
Q1	**Reference**	**Reference**	**Reference**
Q2	− 0.050 (− 0.110, − 0.001) **0.046**	− 0.023 (− 0.079, 0.032) 0.408	− 0.020 (− 0.078, 0.037) 0.478
Q3	− 0.039 (− 0.200, 0.120) 0.640	0.017 (− 0.140, 0.180) 0.838	0.020 (− 0.142, 0.183) 0.796

Thoracic spine-BMD (g/cm^2^)	Model 1^a^	Model 2^b^	Model 3^c^
Dietary approaches to stop hypertension	− 0.060 (− 0.078, − 0.044)** < 0.001**	− 0.044 (− 0.060, − 0.027) < **0.001**	− 0.038 (− 0.057, − 0.020)** < 0.001**
Q1	**Reference**	**Reference**	**Reference**
Q2	− 0.130 (− 0.180, − 0.070) < **0.001**	− 0.090 (− 0.143, − 0.040) **0.001**	− 0.04 (− 0.105, 0.010) 0.110
Q3	− 0.200 (− 0.360, − 0.020) **0.027**	− 0.132 (− 0.300, 0.030) 0.124	− 0.06 (− 0.200, 0.080) 0.407

Pelvic-BMD (g/cm^2^)	Model 1^a^	Model 2^b^	Model 3^c^
Dietary approaches to stop hypertension	− 0.060 (− 0.079, − 0.048) < **0.001**	− 0.040 (− 0.055, − 0.025) < **0.001**	− 0.028 (− 0.043, − 0.013) < **0.001**
Q1	**Reference**	**Reference**	**Reference**
Q2	− 0.110 (− 0.160, 0.040) < **0.001**	− 0.050 (− 0.110, 0.010) 0.090	− 0.024 (− 0.080, 0.030) 0.420
Q3	− 0.300 (− 0.420, − 0.160) < **0.001**	− 0.170 (− 0.310, 0.040) **0.010**	− 0.130 (− 0.290, − 0.010) **0.030**

Significant values are in [bold].

Model 1^b^: no covariates were adjusted; Model 2^c^: adjusted for sex, age, and race; Model 3^d^: adjusted for age, race, sex, education, ratio of family income to poverty, marital status, body mass index, alcohol intake, smoking status, diabetes, and hypertension.

BMD, bone mineral density; 95% CI, 95% confidence interval.

### Subgroup analysis

To conduct a more comprehensive investigation, we performed subgroup analyses and interaction tests to examine the potential impact of a population stratification variable on the observed relationship between DASH and BMD (as shown in Supplementary Material 3). The negative correlations between DASH and BMD remained consistent across specific subgroups. An interaction effect with age was noted in total BMD (p for interaction = 0.04), manifesting a more pronounced negative relationship between DASH and BMD in the older age groups. Among the stratification variables considered, including BMI, gender, diabetes, hypertension, and alcohol, the interaction test was not found to be significant (p for interaction > 0.05, refer to Supplementary Material 3 for specific data). This suggests that the association between DASH and BMD is not influenced by the stratification mentioned above variables.

### Sensitivity analysis

The sensitivity analysis transformed BMD into a Z-score format to facilitate a more comprehensive exploration of its potential linear relationship with DASH diet (Table 4). In the full adjusted model, the β for BMD across the tertiles of DASH was were as follows: total BMD (β = − 0.026, 95% CI: − 0.044, − 0.008), pelvic BMD (β = − 0.028, 95% CI: − 0.043, − 0.013), thoracic spine BMD (β = − 0.038, 95% CI: − 0.057, − 0.020), and lumbar spine BMD (β = − 0.012, 95% CI: − 0.029, 0.004). Overall, the results demonstrated the robustness of the observed correlation between DASH and BMD.

## Discussion

This cross-sectional study encompassed a sample size of 8,486 individuals and aimed to investigate the correlation between the DASH diet and BMD in adults in the United States. The primary objective of this research was to ascertain whether the DASH diet was linked to BMD levels. The findings of this study suggest a negative association between the DASH diet and various aspects of BMD, including total BMD, thoracic BMD, and pelvic BMD. Sensitivity analyses were performed to confirm the findings' robustness. Furthermore, subgroup analyses and interaction tests were conducted, demonstrating that the observed correlation remained unaffected. A previous study reported that changes of about 0.050 g/cm^2^ (equivalent to 4–7% change, depending on the baseline BMD value) are likely to be associated with clinically significant BMD changes^37^. In the context of our findings, the impact of the DASH diet may not be substantial for individuals with healthy BMD. However, for those with borderline BMD, a significant adherence to the DASH diet could potentially push them into the low BMD category.

Previous studies have also suggested a possible negative correlation between the DASH diet and BMD^38^. First, the DASH diet is calcium-rich, but consuming too much calcium can also lead to soft tissue calcification and loss of bone mineral^39^. An experimental study by Doyle and Cashman found that continually feeding a DASH-type diet to rats inhibited bone formation and bone resorption, decreasing BMD^40^. Secondly, a reduced intake of lipids is one of the characteristics of the DASH diet, which leads to lower levels of total cholesterol (TC) and TG in the body^41^. Several studies have shown a positive correlation between TC and TG levels and BMD^42–45^. A cross-sectional study in China showed a positive association between LDL-C and BMD in women^46^. Also, a study from Spain found that BMD was positively associated with total cholesterol and LDL-C^47^. Insights from Hassoon's trial shed light on the connection between DASH, blood osteotriol concentrations, and BMD. Their findings suggest that the impact of the DASH diet on blood osteotriol concentrations could be attributed to the lower fat content, particularly saturated fat^48^. This insight underscores the importance of prudently managing saturated fat intake, particularly for individuals with very high DASH scores. Notably, in the DASH diet, it has been suggested that inorganic nitrate-rich foods can generate nitric oxide (NO) through a non-enzymatic process^49^. The effects of NO on osteoblast (OB) and osteoclast (OC) activity in vivo may vary. Specifically, the induction of pro-inflammatory cytokines, such as tumor necrosis factor-α, interleukin-1β, and interferon-gamma, can promote bone resorption by activating NOS, decreasing BMD^50^.

The precise mechanism underlying the association between the DASH diet and BMD remains uncertain; however, specific evidence suggests a potential negative correlation between the two. The DASH diet has been shown to have a fat-reducing effect^51^. Numerous studies have confirmed the impact of fat on BMD. Specifically, obesity, often accompanied by elevated fat levels, exerts a weight-bearing effect on the skeleton, potentially leading to increased BMD due to this mechanical stimulus^52^. Additionally, it is essential to note that fat is the primary source of aromatase, an enzyme responsible for the synthesis of estrogen^53^. Numerous studies have consistently demonstrated a positive association between estrogen and BMD^54^, indicating that a decrease in fat content will likely result in a decline in BMD.

Consequently, this comprehensive analysis supports the conclusion that implementing the DASH diet is also likely to reduce BMD through its impact on fat reduction. Previous research has indicated that the DASH diet is associated with insufficient fiber consumption^23^. Adequate fiber intake is crucial for maintaining BMD^55^. Investigative studies have demonstrated that fiber intake is protective in preserving BMD^21^ and mitigating bone loss, among other beneficial effects^56^. This phenomenon may be attributed to the alteration of gastrointestinal microorganisms by fiber intake^57^, which in turn influences BMD through the production of short-chain fatty acids (SCFA)^58^. Increased fiber consumption has been found to elevate SCFA levels, thereby promoting calcium absorption^59^. Insufficient dietary fiber consumption resulting from adherence to the DASH diet may contribute to a reduction in BMD via alterations in gastrointestinal microbiota. The DASH diet has resulted in insufficient magnesium intake^23^. Likewise, magnesium intake plays a significant role in maintaining BMD^60^. Inadequate magnesium intake has been linked to a decline in systemic BMD^61^. Animal experiments have demonstrated that animals with magnesium deficiency exhibit delayed development and reduced bone mineral content^62,63^. In both animals and humans, magnesium deficiency leads to reduced secretion of parathyroid hormone^64^ and decreased levels of serum 1,25(OH)2-vitamin D^65^. Insufficiency of these two hormones contributes to impaired bone formation^66^.

Our study's subgroup analysis and interaction test revealed a significant interaction between total BMD in relation to age. Specifically, individuals aged 50 and above exhibited a heightened vulnerability to decreased BMD when adhering to the same DASH diet. One potential factor that may impact BMD is the alteration of nutritional requirements as individuals age. This can be attributed to several reasons. Firstly, individuals over 50 tend to experience a diminished capacity to absorb calcium and vitamin D compared to their younger counterparts^67^. It is worth noting that both calcium and vitamin D have been found to influence BMD positively^68^.

Furthermore, empirical research has demonstrated that supplementing calcium and vitamin D among older adults fails to alter the prevailing pattern of age-related decline in BMD^69^. Consequently, there appears to be a discernible decline in BMD with age among individuals adhering to the DASH diet. Notably, protein consumption is a component of the DASH diet^15^; however, it is observed that digestion tends to be less efficient in older individuals^70^, and the overall protein intake tends to decline with age. A research investigation on sarcopenia elucidated a gradual reduction in muscle mass as individuals age^71^, which was found to have a positive correlation with protein consumption^72^. Additionally, another study demonstrated that muscle mass positively influences the maintenance and enhancement of BMD^73^. Therefore, as the need for protein increases with age, adhering to a long-term DASH diet may contribute to muscle loss, thereby impacting BMD. Hormonal factors were identified as significant contributors in the conducted investigations, wherein it was observed that both testosterone and estrogen levels decrease as individuals age, regardless of gender^74,75^. The insufficiency of testosterone and estrogen is the primary catalyst for reducing BMD among men and women^74,76^. Notably, the DASH diet does not prioritize hormone intake, exacerbating the decline in BMD as individuals age. The DASH diet, which emphasizes the daily consumption of dairy products for their calcium and protein content, may not be suitable for older people^15^. With advancing age, there is a decline in lactase production in the body, resulting in diminished digestion and absorption of lactose among older adults. This leads to lactose intolerance and necessitates reducing dairy intake^77^.

Consequently, as individuals age, the calcium and protein derived from dairy products, essential for maintaining BMD, become less accessible within the DASH population, potentially exacerbating the loss of BMD with increasing age. The reasons mentioned above may contribute to the significant age interaction. Nonetheless, in the case of other variables, including hypertension, diabetes mellitus, BMI, smoking, and gender, the interaction does not exhibit significance. This implies that the relationship between the DASH diet and BMD remains unaffected.

Our study has several noteworthy strengths. Firstly, it uses data from the esteemed NHANES, which has been meticulously weighted to accurately depict the association between the DASH diet and BMD in US adults. Additionally, we have incorporated strategies to address confounding covariates, selecting them based on prior research to ensure the reliability of our results. However, it is important to acknowledge the inherent limitations of our study. Firstly, it is crucial to acknowledge that the study was limited to a cross-sectional design, which unfortunately hinders our ability to definitively establish causality. Secondly, this constraint also restricts our ability to evaluate participants' adherence to the DASH diet and its long-term influence on BMD. Understanding the effects of prolonged adherence to the diet could provide valuable insights into the correlation between the DASH diet and BMD. Additionally, this study found a negative correlation between the DASH diet and BMD. However, determining the specific constituents of the DASH diet that contributed to this correlation proved to be a challenging task. This complexity highlights the need for further refinement in this area. Resolving this issue and achieving improved accuracy in the field of nutrition undoubtedly require additional efforts in future research. Finally, it is of great concern that this study relied on only two 24-h dietary data to make an assessment of DASH, and that a simple average intake over one or two days may not accurately capture usual intake. Future studies, particularly longitudinal cohort or intervention trials, are urgently needed to overcome this limitation.

## Conclusions

This study demonstrates a significant negative correlation between the DASH diet and BMD in various skeletal regions, encompassing total BMD, thoracic spine, and pelvic BMD within the adult population of the United States. Notably, the relationship between the DASH diet and lumbar spine BMD was not found to be significant. Further research is imperative to substantiate these findings.

### Supplementary Information

Supplementary Information.

## Data Availability

The survey data are publicly available on the Internet for data users and researchers throughout the world (www.cdc.gov/nchs/nhanes/).
