# Supplementary material for: The association between dietary approaches to stop hypertension diet and bone mineral density in US adults: evidence from the National Health and Nutrition Examination Survey (2011–2018)
Source: Sci Rep. 2023 Dec 27;13:23043. doi: 10.1038/s41598-023-50423-7 (PMC10754924; doi:10.1038/s41598-023-50423-7)
Supplement: Supplementary file 1 — Supplementary Information. [file 41598_2023_50423_MOESM1_ESM.doc]

**Supplementary Material 1**

**STROBE Statement—Checklist of items that should be included in reports of *cross-sectional studies***

|  | **Item No** | **Recommendation** | **Page No** |
| --- | --- | --- | --- |
| **Title and abstract** | 1 | (*a*) Indicate the study’s design with a commonly used term in the title or the abstract | 1 |
|  |  | (*b*) Provide in the abstract an informative and balanced summary of what was done and what was found | 1-2 |
| **Introduction** | | | |
| Background/rationale | 2 | Explain the scientific background and rationale for the investigation being reported | 3-5 |
| Objectives | 3 | State specific objectives, including any prespecified hypotheses | 5 |
| **Methods** | | | |
| Study design | 4 | Present key elements of study design early in the paper | 5-6 |
| Setting | 5 | Describe the setting, locations, and relevant dates, including periods of recruitment, exposure, follow-up, and data collection | 5-6 |
| Participants | 6 | (*a*) Give the eligibility criteria, and the sources and methods of selection of participants | 6 |
| Variables | 7 | Clearly define all outcomes, exposures, predictors, potential confounders, and effect modifiers. Give diagnostic criteria, if applicable | 7-8 |
| Data sources/ measurement | 8* | For each variable of interest, give sources of data and details of methods of assessment (measurement). Describe comparability of assessment methods if there is more than one group | *5-6* |
| Bias | 9 | Describe any efforts to address potential sources of bias | 8-9 |
| Study size | 10 | Explain how the study size was arrived at | 5 |
| Quantitative variables | 11 | Explain how quantitative variables were handled in the analyses. If applicable, describe which groupings were chosen and why | 7-8 |
| Statistical methods | 12 | (*a*) Describe all statistical methods, including those used to control for confounding | 8-9 |
|  |  | (*b*) Describe any methods used to examine subgroups and interactions | 8-9 |
|  |  | (*c*) Explain how missing data were addressed | 8-9 |
|  |  | (*d*) If applicable, describe analytical methods taking account of sampling strategy | 8-9 |
|  |  | (*e*) Describe any sensitivity analyses | 8-9 |
| **Results** | | | |
| Participants | 13* | (a) Report numbers of individuals at each stage of study—eg numbers potentially eligible, examined for eligibility, confirmed eligible, included in the study, completing follow-up, and analysed | 6 |
|  |  | (b) Give reasons for non-participation at each stage | / |
|  |  | (c) Consider use of a flow diagram | Fig 1 |
| Descriptive data | 14* | (a) Give characteristics of study participants (eg demographic, clinical, social) and information on exposures and potential confounders | 9 |
|  |  | (b) Indicate number of participants with missing data for each variable of interest | 6 |
| Outcome data | 15* | Report numbers of outcome events or summary measures | 6 |
| Main results | 16 | (*a*) Give unadjusted estimates and, if applicable, confounder-adjusted estimates and their precision (eg, 95% confidence interval). Make clear which confounders were adjusted for and why they were included | 9-12 |
|  |  | (*b*) Report category boundaries when continuous variables were categorized | 9-12 |
|  |  | (*c*) If relevant, consider translating estimates of relative risk into absolute risk for a meaningful time period | / |
| Other analyses | 17 | Report other analyses done—eg analyses of subgroups and interactions, and sensitivity analyses | 9-12 |
| **Discussion** | | | |
| Key results | 18 | Summarise key results with reference to study objectives | 12-17 |
| Limitations | 19 | Discuss limitations of the study, taking into account sources of potential bias or imprecision. Discuss both direction and magnitude of any potential bias | 18 |
| Interpretation | 20 | Give a cautious overall interpretation of results considering objectives, limitations, multiplicity of analyses, results from similar studies, and other relevant evidence | 12-17 |
| Generalisability | 21 | Discuss the generalisability (external validity) of the study results | 12-13 |
| **Other information** | | | |
| Funding | 22 | Give the source of funding and the role of the funders for the present study and, if applicable, for the original study on which the present article is based | / |

*Give information separately for exposed and unexposed groups.

**Notes:** An Explanation and Elaboration article discusses each checklist item and gives methodological background and published examples of transparent reporting. The STROBE checklist is best used in conjunction with this article (freely available on the Web sites of PLoS Medicine at http://www.plosmedicine.org/, Annals of Internal Medicine at http://www.annals.org/, and Epidemiology at http://www.epidem.com/). Information on the STROBE Initiative is available at www.strobe-statement.org.

**Supplementary materials 2 : Definition of DASH**

For the purpose of assessing adherence to the DASH dietary pattern, we utilized a DASH score based on the previous work by Gao (1). Essentially, we determined the DASH goals for eight specific nutrients, including total fat, saturated fat, protein, fiber, cholesterol, calcium, magnesium, and potassium (2). While the original DASH study maintained a constant intake of dietary sodium, we included sodium as one of the target nutrients based on the findings of the DASH-Sodium trial (3) and the recommendations of JNC-VI (4). To index the nutrient goals to total energy intake (except for macronutrients), we generated the DASH score by summing up the number of nutrient targets met, with a maximum score of 9. If an individual's intake was between the DASH goal and the nutrient content of the DASH control diet, a score of 0.5 was assigned for that particular nutrient. Additionally, we assessed a categorical outcome to determine the number of individuals who achieved a moderate level of adherence to the DASH dietary approach. Those who met approximately half of the DASH targets (DASH score ≥ 4.5) were classified as DASH accordant.

(1) Gao S. Diet and Exercise—Behavioral Management of Hypertension and Diabetes [dissertation]. Seattle: University of Washington; 2006.

(2) Sacks FM, Obarzanek E, Windhauser MM, et al. Rationale and design of the Dietary Approaches to Stop Hypertension trial (DASH): a multicenter controlled-feeding study of dietary patterns to lower blood pressure. Ann Epidemiol. 1995;5(2):108-118.

(3) The sixth report of the Joint National Committee on prevention, detection, evaluation, and treatment of high blood pressure. Arch Intern Med. 1997;157(21):2413-2446.

| **Nutrient** | **DASH Diet Nutrient Composition^1^** | **DASH Score Target** | **Intermediate Target** |
| --- | --- | --- | --- |
| Saturated fat | 6% of energy | 6% of energy | 11% of energy |
| Total fat | 27% of energy | 27% of energy | 32% of energy |
| Protein | 18% of energy | 18% of energy | 16.5% of energy |
| Cholesterol | 150 mg | 71.4 mg/1000 kcal | 107.1 mg/1000 kcal |
| Fiber | 31 g | 14.8 g/1000 kcal | 9.5 g/1000 kcal |
| Magnesium | 500 mg | 238 mg/1000 kcal | 158 mg/1000 kcal |
| Calcium | 1240 mg | 590 mg/1000 kcal | 402 mg/1000 kcal |
| Potassium | 4700 mg | 2238 mg/1000 kcal | 1534 mg/1000 kcal |
| Sodium^2^ | 2400 mg | 1143 mg/1000 kcal | 1286 mg/1000 kcal |

Abbreviation: DASH, Dietary Approaches to Stop Hypertension trial.

^1^Based on a 2100-kcal diet.^2^Sodium target based on the Sixth Report of the Joint National Committee on Prevention, Detection, Evaluation, and Treatment of High Blood Pressure recommendations.

**Supplementary materials 3 Subgroup analysis for the association of DASH with bone mineral density**

| **TS-BMD g/cm^2^** | ***β* (95%CI)** | ***P*-value** | ***P* for interaction** |
| --- | --- | --- | --- |
| **Age (years)** |  |  | 0.63 |
| <50 | 0.00( 0.00, 0.01) | 0.21 |  |
| ≥50 | -0.01(-0.01,0.01) | 0.14 |  |
|  |  |  |  |
| **Gender** |  |  | 0.35 |
| Male | -0.01(-0.01,0.01) | 0.41 |  |
| Female | -0.01(-0.01,0.01) | 0.03 |  |
|  |  |  |  |
| **BMI((kg/m^2^)** |  |  | 0.12 |
| underweight or normal or overweight (＜30 kg/m^2^) | -0.01(-0.01, 0.00) | 0.001 |  |
| Obese (≥30 kg/m^2^) | -0.01(-0.01, 0.01) | 0.37 |  |
|  |  |  |  |
| **Hypertension** |  |  | 0.98 |
| Yes | -0.01(-0.01,0.01) | 0.01 |  |
| No | 0.00(-0.01,0.00) | 0.08 |  |
|  |  |  |  |
| **Diabetes** |  |  | 0.12 |
| Yes | -0.01(-0.01, 0.01) | 0.47 |  |
| No | -0.01(-0.01, 0.00) | ＜0.001 |  |
|  |  |  |  |
| **Smoking status** |  |  | 0.13 |
| Never | -0.01(-0.01, 0.00) | ＜0.001 |  |
| Former | -0.01(-0.01, 0.00) | 0.04 |  |
| Now | 0.00(-0.01, 0.00) | 0.78 |  |
| **LS-BMD g/cm^2^** | ***β* (95%CI)** | ***P*-value** | ***P* for interaction** |
| **Age (years)** |  |  | 0.06 |
| <50 | 0.00(-0.01, 0.00) | 0.89 |  |
| ≥50 | -0.01(-0.02, 0.00) | 0.13 |  |
|  |  |  |  |
| **Gender** |  |  | 0.66 |
| Male | -0.01(-0.01, 0.00) | 0.24 |  |
| Female | 0.00(-0.01, 0.00) | 0.07 |  |
|  |  |  |  |
| **BMI((kg/m^2^)** |  |  | 0.06 |
| underweight or normal or overweight (＜30 kg/m^2^) | -0.01(-0.01, 0.00) | 0.05 |  |
| Obese (≥30 kg/m^2^) | -0.01(-0.01, 0.00) | 0.61 |  |
|  |  |  |  |
| **Hypertension** |  |  | 0.91 |
| Yes | 0.05(0.03, 0.08) | 0.52 |  |
| No | 0.03(-0.02-0.08) | 0.18 |  |
|  |  |  |  |
| **Diabetes** |  |  | 0.53 |
| Yes | 0.00(-0.01, 0.00) | 0.91 |  |
| No | -0.01(-0.01, 0.00) | 0.53 |  |
|  |  |  |  |
| **Smoking status** |  |  | 0.08 |
| Never | 0.00(-0.01, 0.00) | 0.25 |  |
| Former | -0.01(-0.02, 0.00) | 0.02 |  |
| Now | 0.00(-0.01, 0.00) | 0.78 |  |
| **Pelvic-BMD g/cm^2^** | ***β* (95%CI)** | ***P*-value** | ***P* for interaction** |
| **Age (years)** |  |  | 0.38 |
| <50 | -0.01(-0.01, 0.00) | 0.003 |  |
| ≥50 | -0.01(-0.02, 0.00) | 0.003 |  |
|  |  |  |  |
| **Gender** |  |  | 0.67 |
| Male | -0.01(-0.01, 0.00) | 0.02 |  |
| Female | 0.00(-0.01, 0.00) | 0.02 |  |
|  |  |  |  |
| **BMI((kg/m^2^)** |  |  | 0.29 |
| underweight or normal or overweight (＜30 kg/m^2^) | -0.01(-0.01, 0.00) | <0.001 |  |
| Obese (≥30 kg/m^2^) | 0.00(-0.01, 0.00) | 0.44 |  |
|  |  |  |  |
| **Hypertension** |  |  | 0.45 |
| Yes | -0.01(-0.01, 0.00) | 0.003 |  |
| No | -0.01(-0.01, 0.00) | 0.52 |  |
|  |  |  |  |
| **Diabetes** |  |  | 0.56 |
| Yes | -0.01(-0.02, 0.00) | 0.80 |  |
| No | -0.01(-0.01, 0.00) | 0.002 |  |
|  |  |  |  |
| **Smoking status** |  |  | 0.62 |
| Never | -0.01(-0.01, 0.00) | <0.001 |  |
| Former | -0.01(-0.02, 0.00) | 0.01 |  |
| Now | 0.00(-0.01, 0.00) | 0.92 |  |
| **Total-BMD g/cm^2^** | ***β* (95%CI)** | ***P*-value** | ***P* for interaction** |
| **Age (years)** |  |  | 0.04 |
| <50 | 0.00(-0.01, 0.00) | 0.08 |  |
| ≥50 | -0.01(-0.02, 0.00) | <0.001 |  |
|  |  |  |  |
| **Gender** |  |  | 0.93 |
| Male | -0.01(-0.01, 0.00) | 0.49 |  |
| Female | 0.00(-0.01, 0.00) | 0.70 |  |
|  |  |  |  |
| **BMI((kg/m^2^)** |  |  | 0.63 |
| underweight or normal or overweight (＜30 kg/m^2^) | -0.01(-0.01, 0.00) | 0.22 |  |
| Obese (≥30 kg/m^2^) | 0.00(-0.01, 0.00) | 0.81 |  |
|  |  |  |  |
| **Hypertension** |  |  | 0.91 |
| Yes | -0.01(-0.01, 0.00) | 0.38 |  |
| No | -0.01(-0.01, 0.00) | 0.82 |  |
|  |  |  |  |
| **Diabetes** |  |  | 0.74 |
| Yes | -0.01(-0.01, 0.00) | 0.80 |  |
| No | 0.00(-0.01, 0.00) | 0.45 |  |
|  |  |  |  |
| **Smoking status** |  |  | 0.62 |
| Never | 0.00(-0.01, 0.00) | 0.003 |  |
| Former | -0.01(-0.02, 0.00) | 0.04 |  |
| Now | 0.00(-0.01, 0.00) | 0.22 |  |

**Abbreviation**: **BMI**: body mass index, **LS-BMD**: lumbar spine bone mineral density, **TS-BMD**: thoracic spine bone mineral density

**Note**: adjusted for age, race, sex, education, ratio of family income to poverty, marital status, body mass index, alcohol intake, smoking status, diabetes, hypertension, and average calcium intake
